# Migraine and gastrointestinal disorders in middle and old age: A UK Biobank study

**DOI:** 10.1002/brb3.2291

**Published:** 2021-07-21

**Authors:** Nike Zoe Welander, Gaia Olivo, Claudia Pisanu, Gull Rukh, Helgi Birgir Schiöth, Jessica Mwinyi

**Affiliations:** ^1^ Department of Neuroscience Uppsala University Uppsala Sweden; ^2^ Department of Neurobiology, Care Sciences and Society Karolinska Institutet Stockholm Sweden; ^3^ Department of Biomedical Sciences University of Cagliari Cagliari Italy; ^4^ Institute for Translational Medicine and Biotechnology Sechenov First Moscow State Medical University Moscow Russia

**Keywords:** celiac disease, *Helicobacter pylori*, inflammatory bowel diseases, irritable bowel syndrome, migraine disorders, peptic ulcer

## Abstract

**Introduction:**

Migraine is a prevalent condition causing a substantial level of disability worldwide. Despite this, the pathophysiological mechanisms are not fully understood. Migraine often co‐occurs with gastrointestinal disorders, but the direction of a potential causal link is unclear. The aim of this project was to investigate the associations between migraine and several gastrointestinal disorders in the same cohort in order to determine the relative strengths of these associations.

**Methods:**

This cross‐sectional study examined whether migraine is associated with irritable bowel syndrome (IBS), peptic ulcers, *Helicobacter pylori* (HP) infections, celiac disease, Crohn's disease and ulcerative colitis. Baseline data covering 489,753 UK Biobank participants (migraine group: *n* = 14,180) were analyzed using Pearson's chi‐square tests and adjusted binary logistic regression models.

**Results:**

Migraine was significantly associated with IBS (odds ratio [OR] 2.24, 95% confidence interval [CI] 2.08–2.40, *p* <.001) and peptic ulcers (OR 1.55, 95% CI 1.35–1.77, *p* <.001). Migraine was not associated with HP infection (OR 1.34, 95% CI 1.04–1.73, *p* = .024), celiac disease (OR 1.29, 95% CI 1.04–1.60, *p* = .023), Crohn's disease (OR 1.08, 95% CI 0.80–1.45, *p* = .617) or ulcerative colitis (OR 1.00, 95% CI 0.79–1.27, *p* = .979) after adjusting for multiple testing.

**Conclusions:**

Migraine was associated with IBS and peptic ulcers in this large population‐based cohort. The associations with HP infection, celiac disease, Crohn's disease, and ulcerative colitis did not reach significance, suggesting a weaker link between migraine and autoimmune gastrointestinal conditions or HP infection.

## Graphical abstract

Previous research on migraine and gastrointestinal comorbidity has yielded inconsistent results. This study assessed the associations between migraine and irritable bowel syndrome, peptic ulcers, Helicobacter pylori infections, coeliac disease, Crohn's disease and ulcerative colitis in UK Biobank, a large population‐based cohort. Migraine was strongly associated with irritable bowel syndrome and peptic ulcers, while the remaining associations did not reach significance after controlling for confounding factors and correcting for multiple testing.

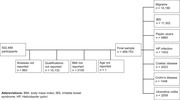

## 1 INTRODUCTION

Migraine is one of the world's most common neurological disorders, affecting 14% of women and 6% of men (Stovner et al., 2007). Even though migraine is a major cause of years lived with disability worldwide (GBD 2016 Headache Collaborators, 2018), its pathophysiological mechanisms are not fully understood. Some insight into factors contributing to migraine may be gained through examining migraine comorbidity. Migraine is associated with multiple gastrointestinal (GI) disorders, including irritable bowel syndrome (IBS) (Cole et al., 2006; Lau et al., 2014; Le Gal et al., 2016), *Helicobacter pylori* (HP) infection (Akbari et al., 2019; Yiannopoulou et al., 2007), celiac disease (Dimitrova et al., 2013; Gabrielli et al., 2003; Lebwohl et al., 2016) and inflammatory bowel disease (IBD) (Chehel Cheraghi et al., 2016; Dimitrova et al., 2013; Moisset et al., 2017). This suggests that migraine may be related to disruptions in the gut–brain axis, which entails that there are bidirectional relationships between neurological and GI symptoms (De Palma et al., 2014). If so, mapping the mechanisms behind the relationships between migraine and GI conditions could facilitate the development of new treatment methods. An important step in this process is to identify which GI conditions share the strongest link to migraine.

IBS is associated with a reduction in perceived quality of life and substantial economic costs (Canavan et al., 2014). The reliance on symptoms rather than biomarkers to establish a diagnosis reflects the fact that multiple mechanisms may underlie IBS. Observed abnormalities include alterations in gut microbiota, low‐grade inflammation and disruptions in the gut serotonergic system (Bellini et al., 2014). The risk of IBS is greater for migraineurs than controls (Lau et al., 2014; Li et al., 2017), and IBS comorbidity increases with headache frequency (Li et al., 2017). Similarly, the risk of migraine is increased in patients with IBS (Cole et al., 2006). Although these results reflect a clear link between IBS and migraine, the mechanisms behind it are elusive.

HP infection is a common cause of peptic ulcers, though many of those infected are free from symptoms (Testerman & Morris, 2014). Studies on migraine and HP infection have obtained mixed results. Some have reported improvements in migraine symptoms upon HP eradication (Faraji et al., 2012; Seyyedmajidi et al., 2016), whereas others have not found any increase in HP prevalence in migraineurs (Lee et al., 2017; Pinessi et al., 2000). However, the largest of these studies was based on 168 migraineurs and 336 controls (Lee et al., 2017). Larger studies may therefore be needed to assess the link between migraine and HP infection.

The lifetime prevalence of peptic ulcers is 5–10% (Lanas & Chan, 2017). Despite the connection between peptic ulcers and HP infection, few have examined the potential comorbidity between peptic ulcers and migraine. A small‐scale study found that duodenal (*n* = 58) but not gastric (*n* = 22) ulcers were associated with migraine (Hormati et al., 2019). Given the modest sample size used in the study, this topic merits further investigation.

Celiac disease is an autoimmune disorder characterized by gluten intolerance (Singh et al., 2018). The condition has been linked to headache in general and migraine in particular. In a meta‐analysis, the pooled prevalence of headache was greater in patients with celiac disease and vice versa (Zis et al., 2018). Moreover, a population‐based study found that patients with celiac disease had more migraine‐related healthcare visits than controls (Lebwohl et al., 2016). In contrast, one study did not find any evidence to support an association between celiac disease and migraine (Inaloo et al., 2011), indicating the need to explore this relationship further.

IBD comprises ulcerative colitis and Crohn's disease, which are characterized by chronic inflammation in the GI tract (Baumgart & Carding, 2007). Small‐scale studies report a high prevalence of migraine among patients with IBD in general (Chehel Cheraghi et al., 2016; Dimitrova et al., 2013; Moisset et al., 2017), but differences emerge when examining the two conditions separately. Ford et al. (2009) found that migraine was more common in patients with Crohn's disease than in patients with ulcerative colitis, whereas Anadol Kelleci et al. (2016) reported that Crohn's disease was associated with tension‐type headache, but not with migraine. These mixed results indicate that larger sample sizes may be needed to examine whether migraine is associated with IBD.

Several mechanisms have been proposed to explain the link between migraine and GI disorders based on the gut–brain axis. One of them is disruptions of the serotonergic system (O'Mahony et al., 2015). Serotonin levels are low between migraine attacks and increase during attacks, which has led to the proposal that migraine may be characterized by chronically low serotonin levels (Hamel, 2007). Interestingly, both IBS and ulcerative colitis have been associated with decreased expression of the serotonin transporter in gut epithelium and low serotonin levels in the colon (Coates et al., 2004). Low serotonin levels may thus constitute a link between migraine and GI conditions.

Other mechanisms linking migraine with GI disorders are increased gut permeability and inflammatory processes (Le Gal et al., 2016; van Hemert et al., 2014). Chronic inflammation in the GI tract, which is characteristic of IBD and celiac disease, has been associated with neurological conditions. Alterations in gut microbiota is a key factor in these processes (Serra et al., 2019). This has led to the idea that treating GI disorders in migraineurs with prebiotics and probiotics may mitigate both GI and migraine symptoms (Doulberis et al., 2017; van Hemert et al., 2014). However, results from randomized controlled trials are inconclusive. While de Roos et al. (2017) did not find any significant effects of probiotic supplements, Martami et al. (2019) reported a decrease in the frequency and severity of migraine attacks after 10 weeks of treatment.

Attempts to compare the strengths of the associations between migraine and different GI conditions have been limited by the heterogeneity of methods used in previous studies (Doulberis et al., 2017). The results from such a comparison might indicate which mechanisms underlie the link between migraine and GI conditions. In addition, research on the potential connection between migraine and peptic ulcers in particular is scarce. Therefore, the aim of this project was to map the associations between migraine and IBS, peptic ulcers, HP infection, celiac disease, Crohn's disease, and ulcerative colitis in middle‐aged and older individuals.

## 2 METHODS

### 2.1 UK Biobank

Data were obtained from the UK Biobank resource, a large prospective cohort study containing health‐related information from more than 500,000 participants. The purpose of the resource is to enable studies concerning diseases of middle and old age. The participants were adults aged between 37 and 69 years at the time of recruitment. Baseline assessments took place between 2006 and 2010 at multiple centers in England, Scotland, and Wales, where the participants signed an electronic consent form. The assessments included physical measurements, questionnaires, and verbal interviews, completed in a standardized fashion (Sudlow et al., 2015). UK Biobank has obtained Research Tissue Bank approval from its Research Ethics Committee (reference 16/NW/0274). This research has been conducted using the UK Biobank Resource under application number 57519. The use of UK Biobank data was approved by the Swedish Ethical Review Authority (2017/198).

### 2.2 Study population

**FIGURE 1:**
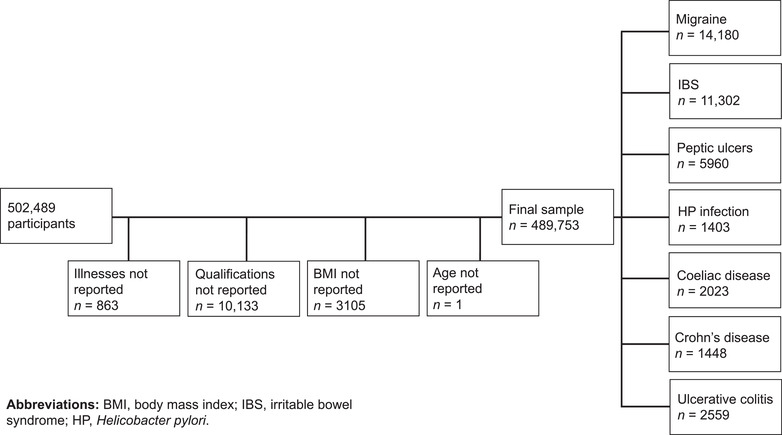
Flowchart of participant inclusion. Participants with missing information on self‐reported illnesses or medications, qualifications, BMI, or age were excluded from the analyses.

In total, 502,489 participants consented to participate in the baseline UK Biobank session. Information was missing for some participants. The affected variables were self‐reported illnesses or medications (*n* = 863), qualifications (*n* = 10,133), BMI (*n* = 3105) and age (*n* = 1). As these variables were used to adjust the analyses (see below), participants with missing information were excluded. In total, 12,736 participants (2.5%) had missing information for one or several of the variables. This yielded a sample size of 489,753 participants (Figure 1). To test whether excluding participants with missing information on qualifications and BMI altered the results, the analyses were rerun based on pooled estimates from five multiple imputation datasets. The results from these analyses did not differ substantially from those presented (see Tables S1 and S2, Supporting Information).

### 2.3 Variables

Diagnoses used in the analyses included migraine, IBS, gastric ulcers, duodenal ulcers, peptic ulcers (unspecified site), HP infection, celiac disease, Crohn's disease, and ulcerative colitis. Gastric, duodenal, and unspecified peptic ulcers were all combined into one variable, referred to as peptic ulcers. The information about medical conditions was collected during verbal interviews with trained staff; this information was thus self‐reported. Self‐reported diagnoses were used because the available codes from the International Statistical Classification of Diseases and Related Health Problems, Tenth Revision (ICD‐10) were obtained from hospital inpatient records. As conditions such as migraine and IBS do not necessarily require hospital care, using ICD‐10 codes would have meant that these conditions had been substantially underreported.

The analyses were adjusted for potentially confounding medical conditions. These included cardiovascular diseases (CVDs) and neurological or GI conditions other than the ones studied. CVDs were included because of their known association with migraine (Schürks et al., 2009). All conditions categorized as “cardiovascular” in UK Biobank were included in the CVD variable. With the exception of migraine, all conditions categorized as “neurological” or “neurological system cancer” were combined in a variable for potentially confounding neurological conditions. Similarly, with the exception of the GI conditions studied, all conditions categorized as “gastrointestinal/abdominal” or “gastrointestinal cancer” were combined in a variable for potentially confounding GI conditions. Lists of the conditions included in these three variables are provided in Table S5, Supporting Information.

The use of nonsteroidal anti‐inflammatory drugs (NSAIDs) to treat migraine could influence a potential relationship between migraine and peptic ulcers. Therefore, the analyses were adjusted for the use of NSAIDs for which migraine is an indication. A list of the drugs included is also provided in Table S5, Supporting Information.

Other variables that were used to adjust the analyses included gender, age, and qualifications (i.e., whether the participants had completed a college or university degree). This information was collected through a touchscreen questionnaire. The variable on qualifications originally contained more categories, but was modified to simplify the models. BMI values were calculated based on height and weight measurements obtained at UK Biobank assessment centers.

### 2.4 Statistical analyses

Unadjusted associations between migraine and GI disorders were investigated using Pearson's chi‐square tests. Subsequently, the associations were tested in binary logistic regression models, adjusting for sociodemographic factors (age, gender, and qualifications), BMI, other neurological or GI conditions, CVDs, and NSAID use, as described above. Because the assumption of linearity, assessed using the Box‐Tidwell test, was violated, age and BMI were converted into ordinal variables. For age, the participants were divided into quintiles. For BMI, the categories underweight/normal weight, overweight and obese were used. Migraine constituted the outcome variable and each disorder of interest was first entered separately. Then, all diagnoses were added to the same model. As the large sample size may render even small associations statistically significant, the dataset was split into random halves analyzed separately (Table S6, Supporting Information).

Statistical analyses were completed using IBM SPSS Statistics version 26.0 (IBM Corp., Armonk, NY, USA) and R version 4.0.2. Bonferroni corrections for multiple comparisons were applied when interpreting the results from the regression models, correcting for the number of independent variables. This yielded a significance level of *p* = .004 (.05 divided by 14) for the final binary logistic regression analyses.

## 3 RESULTS

### 3.1 Background characteristics

Background characteristics and sample prevalence rates of the diseases of interest are provided in Table 1. The total prevalence of migraine was 2.9%. The prevalence was higher among women (4.2%) than men (1.4%). Compared with controls, migraineurs were younger, had a lower BMI and were more likely to have a college or university degree. The percentage of participants with CVD was also lower in the migraine group. The use of NSAIDs indicated for the treatment of migraine was twice as common in the migraine group as in the control group.

**TABLE 1:** Characteristics of participants with and without migraine

Variable	Migraine, *n* (%)	No migraine, *n* (%)	χ^2^ (df)	*p‐*value	Cramér's V	OR (95% CI)
Age (quintiles)						
37–48	3,342 (23.6)	97,687 (20.5)				
49–55	3,591 (25.3)	101,244 (21.3)				
56–60	2,921 (20.6)	93,137 (19.6)	472.5 (4)	< .001	.031	
61–64	2,474 (17.4)	92,224 (19.4)				
65–73	1,852 (13.1)	91,281 (19.2)				
BMI						
< 25	5,513 (38.9)	156,663 (32.9)				
25–29.9	5,460 (38.5)	202,599 (42.6)	220.0 (2)	< .001	.021	
≥ 30	3,207 (22.6)	116,311 (24.5)				
Qualifications						
University degree	5,016 (35.4)	155,371 (32.7)	45.7 (1)	< .001	.010	1.13
Other qualification (ref)	9,164 (64.6)	320,202 (67.3)				(1.09–1.17)
Sex						
Female	11,100 (78.3)	255,694 (53.8)	3336.5 (1)	< .001	.083	3.10
Male (ref)	3,080 (21.7)	219,879 (46.2)				(2.98–3.23)
NSAID use						
Yes	3,973 (28.0)	66,898 (14.1)	2165.5 (1)	< .001	.066	2.38
No (ref)	10,207 (72.0)	408,675 (85.9)				(2.29–2.47)
CVD						
Yes	4,484 (31.6)	174,787 (36.8)	156.2 (1)	< .001	.018	0.80
No (ref)	9,696 (68.4)	300,786 (63.2)				(0.77–0.83)
Other GI conditions						
Yes	2,483 (17.5)	61,788 (13.0)	246.5 (1)	< .001	.022	1.42
No (ref)	11,697 (82.5)	413,785 (87.0)				(1.36–1.49)
Other neurological conditions						
Yes	1,154 (8.1)	21,183 (4.5)	429.3 (1)	< .001	.030	1.90
No (ref)	13,026 (91.9)	454,390 (95.5)				(1.79–2.02)
IBS						
Yes	878 (6.2)	10,424 (2.2)	977.2 (1)	< .001	.045	2.95
No (ref)	13,302 (93.8)	465,149 (97.8)				(2.74–3.16)
Peptic ulcers						
Yes	230 (1.6)	5,730 (1.2)	19.9 (1)	< .001	.006	1.35
No (ref)	13,950 (98.4)	469,843 (98.8)				(1.18–1.54)
HP infection						
Yes	66 (0.5)	1,337 (0.3)	16.4 (1)	< .001	.006	1.66
No (ref)	14,114 (99.5)	474,236 (99.7)				(1.30–2.13)
Celiac disease						
Yes	86 (0.6)	1,937 (0.4)	13.3 (1)	< .001	.005	1.49
No (ref)	14,094 (99.4)	473,636 (99.6)				(1.20–1.85)
Crohn's disease						
Yes	47 (0.3)	1,401 (0.3)	0.6 (1)	.426	.001	1.13
No (ref)	14,133 (99.7)	474,172 (99.7)				(0.84–1.51)
Ulcerative colitis						
Yes	71 (0.5)	2,488 (0.5)	0.1 (1)	.715	.001	0.96
No (ref)	14,109 (99.5)	473,085 (99.5)				(0.76–1.21)

*Note*: Educational categories: University degree = college or university degree; other qualification = A levels/AS levels or equivalent, O levels/GCSEs/CSEs or equivalent, professional qualifications, NVQ/HND/HNC or equivalent, none of the above. Sample sizes: total *n* = 489,753; migraine *n* = 14,180.

Abbreviations: BMI, body mass index; CI, confidence interval; CVD, cardiovascular disease; df, degrees of freedom; GI, gastrointestinal; HP, *Helicobacter pylori*; IBS, irritable bowel syndrome; NSAID, nonsteroidal anti‐inflammatory drugs.

Among the GI disorders studied, IBS was the most common diagnosis (2.3%) in the full sample, followed by peptic ulcers (1.2%), ulcerative colitis (0.5%), celiac disease (0.4%), Crohn's disease (0.3%), and HP infection (0.3%).

### 3.2 Unadjusted analyses

In the unadjusted analyses (Table 1), migraine was significantly more common among participants with IBS, peptic ulcers, HP infection, and celiac disease. In contrast, migraine was as common among participants with IBD (either Crohn's disease or ulcerative colitis) as among controls.

### 3.3 Adjusted analyses

When the analyses were adjusted for potentially confounding factors and each GI condition was entered separately, the associations between migraine and IBS, peptic ulcers, and HP infection were statistically significant (Table 2, models 1–6). The association between migraine and celiac disease was not considered statistically significant after correcting for multiple testing. When all GI conditions were included in the same model (Table 2, model 7), only IBS and peptic ulcers remained significantly associated with migraine. Migraine was neither associated with Crohn's disease nor with ulcerative colitis in any of the adjusted analyses. When splitting the dataset into random halves, HP infection was no longer significantly associated with migraine when analyzed separately, while adjusting for confounding factors (Tables S6a and S6b, Supporting Information). However, the results from the final adjusted model in the supplementary analyses were in accordance with the main analyses, as IBS and peptic ulcers were significantly associated with migraine, having similar odds ratios.

**TABLE 2:** Adjusted associations between gastrointestinal disorders and migraine

Variable	B	SE	OR	95% CI	*p*‐value
Model 1					
IBS	0.81	0.04	**2.25**	(2.09–2.42)	**< .001**
Model 2					
Peptic ulcers	0.49	0.07	**1.63**	(1.42–1.86)	**< .001**
Model 3					
HP infection	0.41	0.13	**1.50**	(1.17–1.93)	**.001**
Model 6					
Celiac disease	0.24	0.11	1.28	(1.03–1.59)	.029
Model 5					
Crohn's disease	0.08	0.15	1.08	(0.81–1.45)	.611
Model 6					
Ulcerative colitis	‐0.01	0.12	1.00	(0.79–1.26)	.973
Model 7					
IBS	0.80	0.04	**2.24**	(2.08–2.40)	**< .001**
Peptic ulcers	0.44	0.07	**1.55**	(1.35–1.77)	**< .001**
HP infection	0.29	0.13	1.34	(1.04–1.73)	.024
Celiac disease	0.25	0.11	1.29	(1.04–1.60)	.023
Crohn's disease	0.08	0.15	1.08	(0.80–1.45)	.617
Ulcerative colitis	0.01	0.12	1.00	(0.79–1.27)	.979

*Note*: Statistically significant results using an α‐level of .004 are in bold. A separate model was run for each gastrointestinal disorder, while adjusting for age, sex, qualifications, body mass index, use of nonsteroidal anti‐inflammatory drugs for which migraine is an indication, comorbidity with other neurological or gastrointestinal diseases than the ones studied, and cardiovascular diseases. Characteristics of model 7: ‐2LL: 121874; Chi‐square: χ^2^ = 6525, df = 18, *p* = <.0005; Nagelkerke *R* ^2^: 5.7%; Hosmer & Lemeshow's test: *p* = .003; classification accuracy: 97.1%. Sample sizes: total *n* = 489,753; migraine *n* = 14,180.

Abbreviations: CI, confidence interval; HP, *Helicobacter pylori*; IBS, irritable bowel syndrome; OR, odds ratio; SE, standard error.

## 4 DISCUSSION

Although previous research has reported a substantial level of comorbidity between migraine and several GI conditions separately, the relative strengths of these associations have remained poorly explored. Based on data from UK Biobank, this study assessed the link between migraine and several important GI conditions at once. These analyses included the association between migraine and peptic ulcers, which, to our knowledge, has only been examined in one small‐scale study in recent years. Migraine was strongly associated with both IBS and peptic ulcers. HP infection and celiac disease were significantly associated with migraine in the unadjusted analyses, but these associations did not remain significant after correcting for multiple testing. In contrast to these observations, Crohn's disease and ulcerative colitis were not associated with migraine in any of the analyses.

The fact that IBS and peptic ulcers were significantly associated with migraine, while celiac disease, Crohn's disease, and ulcerative colitis were not, could be related to differences in pathophysiology. Specifically, migraine was not significantly associated with any of the three autoimmune conditions included in the analyses. Interestingly, associations between migraine and other autoimmune conditions or markers, such as rheumatoid arthritis (Wang et al., 2017) and certain antiphospholipid antibodies (Islam et al., 2017), have previously been reported. This suggests that migraine may be associated with autoimmunity in general. Some findings indicate that there may even be an autoimmune component to migraine pathophysiology (Arumugam & Parthasarathy, 2016). The results from this study do not lend support to that idea. Instead, our results demonstrate that migraine is more strongly associated with IBS and peptic ulcers than with autoimmune GI conditions. While IBS etiology is poorly understood, lifestyle factors appear to play a role. Indeed, patients with IBS are recommended changes in diet and lifestyle by way of treatment (Camilleri, 2018). Similarly, while peptic ulcers are often caused by HP infection, risk factors include high levels of perceived stress in everyday life (Deding et al., 2016). Future studies may wish to evaluate whether the same lifestyle interventions could affect both GI and migraine symptoms in patients with comorbidity.

The strong association between migraine and IBS supports findings from previous research (Cole et al., 2006; Lau et al., 2014; Le Gal et al., 2016). As for migraine and peptic ulcers, the only recent study on this topic reported a significant association between migraine and duodenal ulcers, but not gastric ulcers (Hormati et al., 2019). To ascertain whether the decision to combine gastric and duodenal ulcers into one variable influenced our results, these conditions were also analyzed separately. The odds ratios were similar for the two conditions and both associations were statistically significant in the fully adjusted model (Table S4, Supporting Information). This demonstrates that peptic ulcers in general are associated with migraine, with no apparent difference between gastric and duodenal ulcers.

Previous studies on the prevalence of HP infection in migraineurs have obtained mixed results; while one small‐scale study reported a higher HP infection prevalence in migraineurs (Yiannopoulou et al., 2007), two slightly larger studies did not (Lee et al., 2017; Pinessi et al., 2000). In the present study, the association between migraine and HP infection was significant in both the unadjusted and the adjusted model when HP infection was entered separately. However, when all GI conditions were added to the same adjusted model, the association did not reach statistical significance, using a threshold of *p* = .004. Similarly, there was a statistically significant association between migraine and celiac disease in the unadjusted analyses that did not remain after correcting for multiple testing. If HP infections and celiac disease are indeed linked to migraine, these associations are weaker than those of IBS or peptic ulcers in the current sample. Indeed, the OR of 1.34 obtained for HP infection in the fully adjusted regression model is modest and may not be clinically significant.

Crohn's disease and ulcerative colitis were equally common among controls and migraineurs in our study. This contrasts with several studies reporting associations between IBD and migraine (Chehel Cheraghi et al., 2016; Dimitrova et al., 2013; Moisset et al., 2017). The sample sizes used in these studies were, however, smaller than the IBD group in UK Biobank; the largest study included 203 IBD patients, of whom 83 had probable migraine and 33 strict migraine (Moisset et al., 2017). Nevertheless, the low prevalence of these diagnoses in our sample may have limited our power to detect potential associations. Further studies with ad hoc designs may be needed to assess the link between IBD and migraine.

This study needs to be evaluated in light of its limitations. The prevalence rates of GI disorders in the study population were generally low, ranging from 2.3% for IBS to 0.3% for Crohn's disease and HP infection. In contrast, IBS has a global prevalence of 11% (Lovell & Ford, 2012). As for migraine, the prevalence in this cohort was 2.9%, while the global prevalence of current migraine among adults is 11% (Stovner et al., 2007). These low prevalence rates could partly be explained by a “healthy volunteer” selection bias, as the UK Biobank cohort is not representative of the British population (Fry et al., 2017). This limits the external validity of the study and should be considered when interpreting the results. Similarly, the fact that the UK Biobank cohort only includes individuals of middle and old age limits the external validity of the study.

Another potential limitation is the use of self‐reported illnesses. This method was chosen because conditions such as migraine and IBS do not necessarily require hospital treatment. If ICD‐10 codes obtained from hospital inpatient records had been used, many participants with these conditions would have been treated as controls. Furthermore, relatively few self‐reported migraineurs had HP infection, celiac disease, Crohn's disease, or ulcerative colitis, despite the large sample size of UK Biobank. If ICD‐10 codes had been used, the power to detect potential differences would have decreased substantially. Nevertheless, the lack of significant associations for HP infection, celiac disease and IBD may not solely be explained by the smaller sample sizes for these conditions. As Tables S3 and S4, Supporting Information, demonstrate, the number of participants with self‐reported migraine and duodenal ulcers was similarly low. Despite this, the association between migraine and peptic ulcers remained significant when gastric and duodenal ulcers were analyzed separately.

Another potential concern is that the large sample size of UK Biobank may have resulted in statistically significant associations that are not necessarily clinically relevant. To address this, the dataset was split into random halves analyzed separately (Tables S6a and S6b). When HP infection was analyzed separately, it was no longer significantly associated with migraine in either of the halves, demonstrating that this result from the main analyses should be interpreted with caution. Nevertheless, the results from the final model for each of these halves were in accordance with the results from the main analyses, with similar odds ratios.

The study is also limited by the cross‐sectional nature of the analyses. Because of this, the direction of the associations between migraine and the GI conditions of interest could not be assessed. Future studies may wish to employ a prospective study design with larger sample sizes to explore the direction of these associations.

## 5 CONCLUSIONS

In this study, IBS and peptic ulcers were significantly associated with migraine after adjusting for confounding factors, while HP infection, celiac disease, Crohn's disease, and ulcerative colitis were not. This indicates that the link between migraine and autoimmune GI conditions is weaker than that of migraine and IBS and peptic ulcers.

To our knowledge, the strong association between migraine and peptic ulcers has not previously been reported in a large cohort in recent times.

## CONFLICT OF INTEREST

The authors report no conflicts of interest in this work.

## AUTHORS CONTRIBUTIONS

Jessica Mwinyi and Helgi Birgir Schiöth designed the study. Nike Zoe Welander analyzed the data and drafted the manuscript. Gaia Olivo, Claudia Pisanu, Gull Rukh, Jessica Mwinyi and Helgi Birgir Schiöth critically assessed the analyses and revised the manuscript.

### PEER REVIEW

The peer review history for this article is available at https://publons.com/publon/10.1002/brb3.2291.

## Supporting information

Supporting Information

Supporting Information

Supporting Information

Supporting Information

Supporting Information

Supporting Information

## Data Availability

The data that support the findings of this study are available from the UK Biobank Resource (https://www.ukbiobank.ac.uk). Restrictions apply to the availability of these data, which were used under license for this study.
