# Supplementary material for: Migraine and gastrointestinal disorders in middle and old age: A UK Biobank study
Source: Brain Behav. 2021 Jul 21;11(8):e2291. doi: 10.1002/brb3.2291 (PMC8413796; doi:10.1002/brb3.2291)
Supplement: Supplementary file 1 — Supporting Information [file BRB3-11-e2291-s001.docx]

**Table 1** Characteristics of participants with and without migraine using pooled estimates from five multiply-imputed datasets

| **Variable** | **Migraine,**  ***n* (%)** | **No migraine, *n* (%)** | **Combined *D_2_* (df, df2)** | ***p-*value** | **Cramér’s V** | **OR**  **(95% CI)** |
| --- | --- | --- | --- | --- | --- | --- |
| Age (quintiles)  37–48  49–55  56–60  61–64  65–73 | 3,397 (23.6)  3,644 (25.3)  2,967 (20.6)  2,507 (17.4)  1,894 (13.1) | 100,301 (20.6)  103,698 (21.2)  95,384 (19.5)  94,585 (19.4)  94,111 (19.3) | 120.3 (4, 942,276) | < .001 | .031 |  |
| BMI  < 25  25–29.9  ≥ 30 | 5,599 (38.9)  5,546 (38.5)  3,264 (22.7) | 160,367 (32.9)  207,904 (42.6)  119,808 (24.5) | 113.9 (2, 224,984) | < .001 | .021 |  |
| Qualifications  University degree  Other qualification (ref) | 5,096 (35.4)  9,312 (64.6) | 159,290 (32.6)  328,789 (67.4) | 46.2 (1, 4796) | < .001 | .010 | 1.13  (1.09–1.17) |
| Sex  Female  Male (ref) | 11,277 (78.3)  3,131 (21.7) | 262,098 (53.7)  225,981 (46.3) | 3402.3 (1, 3,989,306) | < .001 | .082 | 3.02  (2.90–3.14) |
| NSAID use  Yes  No (ref) | 4,031 (28.0)  10,377 (72.0) | 68,224 (14.0)  419,856 (86.0) | 2223.7 (1, 1,025,552) | < .001 | .067 | 2.39  (2.30–2.48) |
| CVD  Yes  No (ref) | 4,568 (31.7)  9,840 (68.3) | 180,034 (36.9)  308,045 (63.1) | 161.6 (1, 18,638,696) | < .001 | .018 | 0.79  (0.77–0.82) |
| Other GI conditions  Yes  No (ref) | 2,528 (17.5)  11,880 (82.5) | 63,145 (13.0)  424,935 (87.1) | 260.8 (1, 223,563) | < .001 | .023 | 1.43  (1.37­–1.50) |
| Other neurological conditions  Yes  No (ref) | 1,182 (8.2)  13,226 (91.8) | 22,065 (4.5)  466,015 (95.5) | 428.7 (1, 154,813) | < .001 | .029 | 1.89  (1.78–2.01) |
| IBS  Yes  No (ref) | 891 (6.2)  13,517 (93.8) | 10,617 (2.2)  477,462 (97.8) | 977.7 (1, 54,351) | < .001 | .045 | 2.97  (2.76–3.18) |
| Peptic ulcers  Yes  No (ref) | 236 (1.6)  14,172 (98.4) | 5,901 (1.2)  482,178 (98.8) | 21.6 (1, 888,093) | < .001 | .007 | 1.36  (1.19–1.55) |
| HP infection  Yes  No (ref) | 66 (0.5)  14,342 (99.5) | 1,352 (0.3)  486,728 (99.7) | 16.5 (1, 94,496) | < .001 | .006 | 1.66  (1.30–2.13) |
| Coeliac disease  Yes  No (ref) | 86 (0.6)  14,322 (99.4) | 1,964 (0.4)  486,115 (99.6) | 13.1 (1, 237,958) | < .001 | .005 | 1.49  (1.20–1.85) |
| Crohn’s disease  Yes  No (ref) | 47 (0.3)  14,361 (99.7) | 1,431 (0.3)  486,648 (99.7) | 0.6 (1, 166,837) | .456 | .001 | 1.12  (0.83–1.49) |
| Ulcerative colitis  Yes  No (ref) | 71 (0.5)  14,337 (99.5) | 2,529 (0.5)  485,550 (99.5) | 0.2 (1, 261,942) | .695 | .001 | 0.95  (0.75–1.21) |

**Notes:** Qualifications: university degree = college or university degree; other qualification = A levels/AS levels or equivalent, O levels/GCSEs/CSEs or equivalent, professional qualifications, NVQ/HND/HNC or equivalent, none of the above. Sample sizes: total *n* = 502,488; migraine *n* = 14,408.

**Abbreviations:** df, degrees of freedom; CI, confidence interval; BMI, body mass index; NSAID, nonsteroidal anti-inflammatory drugs; CVD, cardiovascular disease; GI, gastrointestinal; IBS, irritable bowel syndrome; HP, *Helicobacter pylori*.
