# Supplementary material for: Migraine and gastrointestinal disorders in middle and old age: A UK Biobank study
Source: Brain Behav. 2021 Jul 21;11(8):e2291. doi: 10.1002/brb3.2291 (PMC8413796; doi:10.1002/brb3.2291)
Supplement: Supplementary file 2 — Supporting Information [file BRB3-11-e2291-s003.docx]

| **Variable** | **B** | **SE** | **OR** | **95% CI** | ***p*-value** |
| --- | --- | --- | --- | --- | --- |
| Model 1  IBS | 0.81 | 0.04 | **2.25** | (2.09–2.42) | **< .001** |
| Model 2  Peptic ulcers | 0.49 | 0.07 | **1.64** | (1.43–1.87) | **< .001** |
| Model 3  HP infection | 0.41 | 0.13 | **1.51** | (1.17–1.94) | **.001** |
| Model 4  Coeliac disease | 0.24 | 0.11 | 1.27 | (1.02–1.58) | .033 |
| Model 5  Crohn’s disease | 0.07 | 0.15 | 1.07 | (0.80–1.43) | .656 |
| Model 6  Ulcerative colitis | -0.01 | 0.12 | 0.99 | (0.78–1.26) | .946 |
| Model 7  IBS  Peptic ulcers  HP infection  Coeliac disease  Crohn’s disease  Ulcerative colitis | 0.81  0.45  0.29  0.25  0.07  -0.01 | 0.04  0.07  0.13  0.11  0.15  0.12 | **2.25**  **1.56**  1.34  1.28  1.07  1.00 | (2.09–2.41)  (1.37–1.79)  (1.04–1.73)  (1.03–1.60)  (0.80–1.43)  (0.79–1.27) | **< .001**  **< .001**  .024  .026  .664  .991 |

**Supplementary table 2** Adjusted associations between gastrointestinal disorders and migraine using pooled estimates from five multiply-imputed datasets

**Notes:** Statistically significant results using an α-level of .004 are in bold. A separate model was run for each gastrointestinal disorder, while adjusting for age, sex, qualifications, body mass index, use of nonsteroidal anti-inflammatory drugs for which migraine is an indication, comorbidity with other neurological or gastro-intestinal diseases than the ones studied, and cardiovascular diseases. Characteristics of the final (pooled) model: -2LL: 124066; Chi-square: 𝜒^2^ = 6684, df = 18, p = <.001; Nagelkerke R^2^: 5.8%; Hosmer & Lemeshow's test: *p* = .002; classification accuracy: 97.1%. Sample sizes: total *n* = 502,488; migraine *n* = 14,408.

**Abbreviations:** SE, standard error; OR, odds ratio; CI, confidence interval; IBS, irritable bowel syndrome; HP, *Helicobacter pylori*.
