# Supplementary material for: Migraine and gastrointestinal disorders in middle and old age: A UK Biobank study
Source: Brain Behav. 2021 Jul 21;11(8):e2291. doi: 10.1002/brb3.2291 (PMC8413796; doi:10.1002/brb3.2291)
Supplement: Supplementary file 3 — Supporting Information [file BRB3-11-e2291-s005.docx]

| **Variable** | **Migraine,**  ***n* (%)** | **No migraine,**  ***n* (%)** | **χ^2^ (df)** | ***p-*value** | **OR**  **(95% CI)** | **Cramér’s V** |
| --- | --- | --- | --- | --- | --- | --- |
| Gastric ulcers  Yes  No (ref) | 138 (1.0)  14,042 (99.0) | 3,406 (0.7)  472,172 (99.3) | 12.7 (1) | 3.730 × 10^-4^ | 1.36  (1.15–1.62) | .005 |
| Duodenal ulcers  Yes  No (ref) | 77 (0.5)  14,103 (99.5) | 1,908 (0.4)  473,665 (99.6) | 6.9 (1) | .009 | 1.36  (1.08–1.70) | .004 |

**Supplementary table 3** Sample prevalence of gastric and duodenal ulcers

**Notes:** Sample sizes: total *n* = 489,753; migraine *n* = 14,180.

**Abbreviations:** df, degrees of freedom; OR, odds ratio; CI, confidence interval.
