# Supplementary material for: Migraine and gastrointestinal disorders in middle and old age: A UK Biobank study
Source: Brain Behav. 2021 Jul 21;11(8):e2291. doi: 10.1002/brb3.2291 (PMC8413796; doi:10.1002/brb3.2291)
Supplement: Supplementary file 4 — Supporting Information [file BRB3-11-e2291-s002.docx]

| **Variable** | **B** | **SE** | **OR** | **95% CI** | ***p*-value** |
| --- | --- | --- | --- | --- | --- |
| Model 1  Gastric ulcers | 0.47 | 0.09 | **1.59** | (1.34–1.90) | **< .001** |
| Model 2  Duodenal ulcers | 0.52 | 0.12 | **1.69** | (1.34–2.13) | **< .001** |
| Model 3  IBS  Gastric ulcers  Duodenal ulcers  HP infection  Coeliac disease  Crohn’s disease  Ulcerative colitis | 0.80  0.42  0.45  0.30  0.25  0.08  0.01 | 0.04  0.09  0.12  0.13  0.11  0.15  0.12 | **2.24**  **1.51**  **1.56**  1.34  1.29  1.08  1.00 | (2.08–2.40)  (1.27–1.80)  (1.24–1.97)  (1.04–1.73)  (1.04–1.61)  (0.80–1.45)  (0.79–1.27) | **< .001**  **< .001**  **< .001**  .022  .023  .614  .980 |

**Supplementary table 4** Adjusted associations between gastrointestinal disorders and migraine with gastric and duodenal ulcers displayed separately.

**Notes:** Statistically significant results are in bold. In model 3, a *p*-value below .003 is considered statistically significant (.05/15 = .003). A separate model was run for gastric and duodenal ulcers, respectively, while adjusting for age, sex, qualifications, body mass index, use of nonsteroidal anti-inflammatory drugs for which migraine is an indication, comorbidity with other neurological or gastrointestinal diseases than the ones studied and cardiovascular disease. Characteristics of the final model: -2LL: 121877; Chi-square: 𝜒^2^ = 6522, df = 19, p = <.0005; Nagelkerke R^2^: 5.7%; Hosmer & Lemeshow's test: *p* = .002; classification accuracy: 97.1%. Total sample size: 489,753.

**Abbreviations:** SE, standard error; OR, odds ratio; CI, confidence interval; IBS, irritable bowel syndrome; HP, *Helicobacter pylori*.
