# Supplementary material for: Migraine and gastrointestinal disorders in middle and old age: A UK Biobank study
Source: Brain Behav. 2021 Jul 21;11(8):e2291. doi: 10.1002/brb3.2291 (PMC8413796; doi:10.1002/brb3.2291)
Supplement: Supplementary file 5 — Supporting Information [file BRB3-11-e2291-s004.docx]

**Supplementary Table 5** Lists of medical conditions and medications included in covariates

| **Neurological conditions other than migraine** | |
| --- | --- |
| UKB 20002 code | Name |
| 1244 | infection of nervous system |
| 1245 | brain abscess/intracranial abscess |
| 1246 | encephalitis |
| 1247 | meningitis |
| 1248 | spinal abscess |
| 1249 | cranial nerve problem/palsy |
| 1250 | bell's palsy/facial nerve palsy |
| 1523 | trigeminal neuralgia |
| 1251 | spinal cord disorder |
| 1252 | paraplegia |
| 1524 | spina bifida |
| 1254 | peripheral nerve disorder |
| 1255 | peripheral neuropathy |
| 1256 | acute infective polyneuritis/guillain-barre syndrome |
| 1257 | trapped nerve/compressed nerve |
| 1468 | diabetic neuropathy/ulcers |
| 1258 | chronic/degenerative neurological problem |
| 1259 | motor neurone disease |
| 1260 | myasthenia gravis |
| 1261 | multiple sclerosis |
| 1262 | parkinsons disease |
| 1263 | dementia/alzheimers/cognitive impairment |
| 1397 | other demyelinating disease (not multiple sclerosis) |
| 1264 | epilepsy |
| 1265 | migraine |
| 1433 | cerebral palsy |
| 1434 | other neurological problem |
| 1436 | headaches (not migraine) |
| 1437 | myasthenia gravis 2 |
| 1525 | benign / essential tremor |
| 1526 | polio / poliomyelitis |
| 1659 | meningioma / benign meningeal tumour |
| 1683 | benign neuroma |
| 1240 | neurological injury/trauma |
| 1266 | head injury |
| 1267 | spinal injury |
| 1394 | peripheral nerve injury |
| **Neurological system cancers** | |
| UKB 20001 code | Name |
| 1029 | peripheral nerve/autonomic nerve cancer |
| 1030 | eye and/or adnexal cancer |
| 1075 | retinoblastoma |
| 1031 | meningeal cancer / malignant meningioma |
| 1032 | brain cancer / primary malignant brain tumour |
| 1033 | spinal cord or cranial nerve cancer |

| **Gastrointestinal/abdominal conditions other than the ones studied** | |
| --- | --- |
| UKB 20002 code | Name |
| 1134 | oesophageal disorder |
| 1138 | gastro-oesophageal reflux (gord) / gastric reflux |
| 1139 | oesophagitis/barretts oesophagus |
| 1140 | oesophageal stricture |
| 1141 | oesophageal varicies |
| 1474 | hiatus hernia |
| 1135 | stomach disorder |
| 1142 | gastric/stomach ulcers |
| 1143 | gastritis/gastric erosions |
| 1501 | pyloric stenosis |
| 1154 | irritable bowel syndrome |
| 1456 | malabsorption/coeliac disease |
| 1457 | duodenal ulcer |
| 1458 | diverticular disease/diverticulitis |
| 1459 | colitis/not crohns or ulcerative colitis |
| 1460 | rectal or colon adenoma/polyps |
| 1461 | inflammatory bowel disease |
| 1462 | crohns disease |
| 1463 | ulcerative colitis |
| 1502 | appendicitis |
| 1503 | anal problem |
| 1504 | anal fissure |
| 1505 | haemorrhoids / piles |
| 1599 | constipation |
| 1600 | bowel / intestinal perforation |
| 1601 | bowel / intestinal infarction |
| 1602 | bowel / intestinal obstruction |
| 1603 | rectal prolapse |
| 1136 | liver/biliary/pancreas problem |
| 1155 | hepatitis |
| 1156 | infective/viral hepatitis |
| 1578 | hepatitis a |
| 1579 | hepatitis b |
| 1580 | hepatitis c |
| 1581 | hepatitis d |
| 1582 | hepatitis e |
| 1157 | non-infective hepatitis |
| 1158 | liver failure/cirrhosis |
| 1506 | primary biliary cirrhosis |
| 1604 | alcoholic liver disease / alcoholic cirrhosis |
| 1159 | bile duct disease |
| 1160 | bile duct obstruction/ascending cholangitis |
| 1475 | sclerosing cholangitis |
| 1161 | gall bladder disease |
| 1162 | cholelithiasis/gall stones |
| 1163 | cholecystitis |
| 1164 | pancreatic disease |
| 1165 | pancreatitis |
| 1507 | haemochromatosis |
| 1508 | jaundice (unknown cause) |
| 1137 | other abdominal problem |
| 1190 | peritonitis |
| 1191 | gastrointestinal bleeding |
| 1400 | peptic ulcer |
| 1509 | gastroenteritis/dysentry |
| 1510 | dyspepsia / indigestion |
| 1511 | abdominal hernia |
| 1512 | umbilical hernia |
| 1513 | inguinal hernia |
| 1605 | femoral hernia |
| 1606 | incisional hernia |
| 1442 | helicobacter pylori |
| 1514 | cystitis |
| **Gastrointestinal cancers** | |
| UKB 20001 code | Name |
| 1017 | oesophageal cancer |
| 1018 | stomach cancer |
| 1019 | small intestine/small bowel cancer |
| 1020 | large bowel cancer/colorectal cancer |
| 1022 | colon cancer/sigmoid cancer |
| 1086 | appendix cancer |
| 1023 | rectal cancer |
| 1021 | anal cancer |
| 1024 | liver/hepatocellular cancer |
| 1025 | gallbladder/bile duct cancer |
| 1026 | pancreas cancer |
| 1088 | malignant insulinoma |

| **CVD conditions** | |
| --- | --- |
| UKB 20002 code | Name |
| 1065 | hypertension |
| 1072 | essential hypertension |
| 1073 | gestational hypertension/pre-eclampsia |
| 1066 | heart/cardiac problem |
| 1074 | angina |
| 1075 | heart attack/myocardial infarction |
| 1076 | heart failure/pulmonary odema |
| 1077 | heart arrhythmia |
| 1471 | atrial fibrillation |
| 1483 | atrial flutter |
| 1484 | wolff parkinson white / wpw syndrome |
| 1485 | irregular heart beat |
| 1486 | sick sinus syndrome |
| 1487 | svt / supraventricular tachycardia |
| 1078 | heart valve problem/heart murmur |
| 1584 | mitral valve disease |
| 1488 | mitral valve prolapse |
| 1489 | mitral stenosis |
| 1585 | mitral regurgitation / incompetence |
| 1586 | aortic valve disease |
| 1490 | aortic stenosis |
| 1587 | aortic regurgitation / incompetence |
| 1079 | cardiomyopathy |
| 1588 | hypertrophic cardiomyopathy (hcm / hocm) |
| 1080 | pericardial problem |
| 1589 | pericarditis |
| 1590 | pericardial effusion |
| 1426 | myocarditis |
| 1479 | rheumatic fever |
| 1081 | stroke |
| 1086 | subarachnoid haemorrhage |
| 1491 | brain haemorrhage |
| 1583 | ischaemic stroke |
| 1082 | transient ischaemic attack (tia) |
| 1083 | subdural haemorrhage/haematoma |
| 1425 | cerebral aneurysm |
| 1067 | peripheral vascular disease |
| 1087 | leg claudication/ intermittent claudication |
| 1088 | arterial embolism |
| 1492 | aortic aneurysm |
| 1591 | aortic aneurysm rupture |
| 1592 | aortic dissection |
| 1068 | venous thromboembolic disease |
| 1093 | pulmonary embolism +/- dvt |
| 1094 | deep venous thrombosis (dvt) |
| 1473 | high cholesterol |
| 1493 | other venous/lymphatic disease |
| 1494 | varicose veins |
| 1495 | lymphoedema |
| 1593 | varicose ulcer |

| **NSAIDs** | |
| --- | --- |
| UKB 20003 code | Name |
| 1140871310 | ibuprofen |
| 1140911754 | anadin tablet |
| 1141153134 | anadin ibuprofen 200mg tablet |
| 1140911758 | hedex tablet |
| 1140878030 | ibuprofen+codeine phosphate |
| 1140910496 | propionic acid-ibuprofen |
| 1141157412 | ibuprofen product |
| 1140871374 | brufen 200mg tablet |
| 1141191742 | calprofen 100mg/5ml s/f oral suspension |
| 1140884488 | diclofenac |
| 1140871168 | voltarol 25mg e/c tablet |
| 1140871542 | mefenamic acid |
| 1140928840 | tolfenamic acid |
| 1140928844 | clotam 200mg capsule |
| 1140871236 | flurbiprofen |
