# Supplementary material for: Migraine and gastrointestinal disorders in middle and old age: A UK Biobank study
Source: Brain Behav. 2021 Jul 21;11(8):e2291. doi: 10.1002/brb3.2291 (PMC8413796; doi:10.1002/brb3.2291)
Supplement: Supplementary file 6 — Supporting Information [file BRB3-11-e2291-s006.docx]

**Supplementary table 6a** Adjusted associations between gastrointestinal disorders and migraine in a random half of the dataset

| **Variable** | **B** | **SE** | **OR** | **95% CI** | ***p*-value** |
| --- | --- | --- | --- | --- | --- |
| Model 1  IBS | 0.82 | 0.05 | **2.28** | (2.06–2.53) | **< .001** |
| Model 2  Peptic ulcers | 0.51 | 0.10 | **1.66** | (1.37–2.01) | **< .001** |
| Model 3  HP infection | 0.35 | 0.18 | 1.43 | (0.99–2.03) | .051 |
| Model 6  Coeliac disease | 0.36 | 0.15 | 1.44 | (1.08–1.92) | .013 |
| Model 5  Crohn’s disease | 0.30 | 0.19 | 1.35 | (0.93–1.95) | .111 |
| Model 6  Ulcerative colitis | -0.16 | 0.18 | 0.85 | (0.59–1.22) | .369 |
| Model 7  IBS  Peptic ulcers  HP infection  Coeliac disease  Crohn’s disease  Ulcerative colitis | 0.82  0.46  0.22  0.38  0.29  -0.16 | 0.05  0.10  0.18  0.15  0.19  0.18 | **2.26**  **1.59**  1.24  1.46  1.34  0.85 | (2.04–2.51)  (1.31–1.92)  (0.87–1.78)  (1.09–1.94)  (0.93–1.94)  (0.60–1.22) | **< .001**  **< .001**  .237  .011  .121  .381 |

**Notes:** Statistically significant results using an α-level of .004 are in bold. A separate model was run for each gastrointestinal disorder, while adjusting for age, sex, qualifications, body mass index, use of nonsteroidal anti-inflammatory drugs for which migraine is an indication, comorbidity with other neurological or gastrointestinal diseases than the ones studied and cardiovascular diseases. Characteristics of model 7: -2LL: 61,158; Chi-square: 𝜒^2^ = 3,216, df = 18, *p* = <.0005; Nagelkerke R^2^: 5.6%; Hosmer & Lemeshow's test: *p* = .080; classification accuracy: 97.1%. Sample sizes: total *n* = 244,873; migraine *n* = 7,115.

**Abbreviations:** SE, standard error; OR, odds ratio; CI, confidence interval; IBS, irritable bowel syndrome; HP, *Helicobacter pylori*.

**Supplementary table 6b** Adjusted associations between gastrointestinal disorders and migraine in a random half of the dataset

| **Variable** | **B** | **SE** | **OR** | **95% CI** | ***p*-value** |
| --- | --- | --- | --- | --- | --- |
| Model 1  IBS | 0.80 | 0.05 | **2.22** | (2.00–2.46) | **< .001** |
| Model 2  Peptic ulcers | 0.46 | 0.10 | **1.59** | (1.31–1.92) | **< .001** |
| Model 3  HP infection | 0.46 | 0.18 | 1.59 | (1.11–2.27) | .011 |
| Model 6  Coeliac disease | 0.10 | 0.17 | 1.10 | (0.79–1.54) | .570 |
| Model 5  Crohn’s disease | -0.23 | 0.25 | 0.80 | (0.49–1.29) | .356 |
| Model 6  Ulcerative colitis | 0.14 | 0.16 | 1.15 | (0.84–1.58) | .393 |
| Model 7  IBS  Peptic ulcers  HP infection  Coeliac disease  Crohn’s disease  Ulcerative colitis | 0.79  0.41  0.37  0.11  -0.22  0.15 | 0.05  0.10  0.18  0.17  0.25  0.16 | **2.21**  **1.51**  1.44  1.11  0.80  1.16 | (1.99–2.47)  (1.25–1.83)  (1.01–2.07)  (0.79–1.56)  (0.49–1.30)  (0.84–1.60) | **< .001**  **< .001**  .044  .536  .365  .362 |

**Notes:** Statistically significant results using an α-level of .004 are in bold. A separate model was run for each gastrointestinal disorder, while adjusting for age, sex, qualifications, body mass index, use of nonsteroidal anti-inflammatory drugs for which migraine is an indication, comorbidity with other neurological or gastrointestinal diseases than the ones studied and cardiovascular diseases. Characteristics of model 7: -2LL: 60,699; Chi-square: 𝜒^2^ = 3,325, df = 18, *p* = <.0005; Nagelkerke R^2^: 5.9%; Hosmer & Lemeshow's test: *p* = .017; classification accuracy: 97.1%. Sample sizes: total *n* = 244,885; migraine *n* = 7,065.

**Abbreviations:** SE, standard error; OR, odds ratio; CI, confidence interval; IBS, irritable bowel syndrome; HP, *Helicobacter pylori*.
